# Altered Glycosylation in Progression and Management of Bladder Cancer

**DOI:** 10.3390/molecules28083436

**Published:** 2023-04-13

**Authors:** Magdalena Wilczak, Magdalena Surman, Małgorzata Przybyło

**Affiliations:** 1 Department of Glycoconjugate Biochemistry, Faculty of Biology, Institute of Zoology and Biomedical Research, Jagiellonian University, Gronostajowa 9 Street, 30-387 Krakow, Poland; magdalena.wilczak@doctoral.uj.edu.pl (M.W.); magdalena.surman@uj.edu.pl (M.S.); 2 Doctoral School of Exact and Natural Sciences, Jagiellonian University, Prof. S. Łojasiewicza 11 Street, 30-348 Krakow, Poland

**Keywords:** bladder cancer, extracellular vesicles, glycosaminoglycans, Lewis’s antigen, mucins, N-glycosylation, O-glycosylation, T antigen, Tn antigen, tumor glycosylation

## Abstract

Bladder cancer (BC) is the 10th most common malignancy worldwide, with an estimated 573,000 new cases and 213,000 deaths in 2020. Available therapeutic approaches are still unable to reduce the incidence of BC metastasis and the high mortality rates of BC patients. Therefore, there is a need to deepen our understanding of the molecular mechanisms underlying BC progression to develop new diagnostic and therapeutic tools. One such mechanism is protein glycosylation. Numerous studies reported changes in glycan biosynthesis during neoplastic transformation, resulting in the appearance of the so-called tumor-associated carbohydrate antigens (TACAs) on the cell surface. TACAs affect a wide range of key biological processes, including tumor cell survival and proliferation, invasion and metastasis, induction of chronic inflammation, angiogenesis, immune evasion, and insensitivity to apoptosis. The purpose of this review is to summarize the current information on how altered glycosylation of bladder cancer cells promotes disease progression and to present the potential use of glycans for diagnostic and therapeutic purposes.

## 1. Introduction

Bladder cancer (BC) is the 10th most common malignancy worldwide, which accounts for 3% of global cancer diagnoses. The highest incidence rate of BC is observed in men in Southern and Western Europe and in North America [1]. According to GLOBOCAN, the estimated number of new BC cases in 2020 reached 573,000, and there were 213,000 new BC-related deaths [2]. BC is a highly heterogeneous cancer with a spectrum ranging from low-grade non-invasive tumors with good prognosis but high recurrence rate up to high-grade muscle-invasive tumors with a poor prognosis. Histologically, BC is classified into two categories: urothelial, which is diagnosed in more than 90% of cases, and non-urothelial, including squamous cell carcinoma, adenocarcinoma, and small cell carcinoma [3,4]. Based on invasiveness, BC is classified into non-muscle-infiltrating bladder cancer (NMIBC, 70% of cases) and muscle-infiltrating bladder cancer (MIBC, 30% of cases). Different therapeutic strategies for BC are applied based on the BC category. In the case of NMIBC, transurethral resection of bladder tumor (TURBT) is the most common strategy, followed by intravesical chemotherapy or immunotherapy, while in MIBC, cystectomy is used with a combination of chemotherapeutic drugs [5]. Unfortunately, current therapeutic approaches do not reduce high mortality rates in BC due to its frequent recurrence after TURBT and the possibility of redevelopment to more invasive and metastatic tumors [6,7]. Therefore, more attention should be devoted to a better understanding of the mechanisms underlying BC progression to establish new diagnostic and therapeutic tools in the future.

The aim of this review article is to summarize the current information on how altered glycosylation of BC cells promotes disease progression and to present the potential use of glycans for diagnostic and therapeutic purposes. Additionally, recent findings on the glycosylation status of BC-derived extracellular vesicles will be presented.

## 2. Alteration in Glycosylation Observed in Cancer

**Figure 1:**
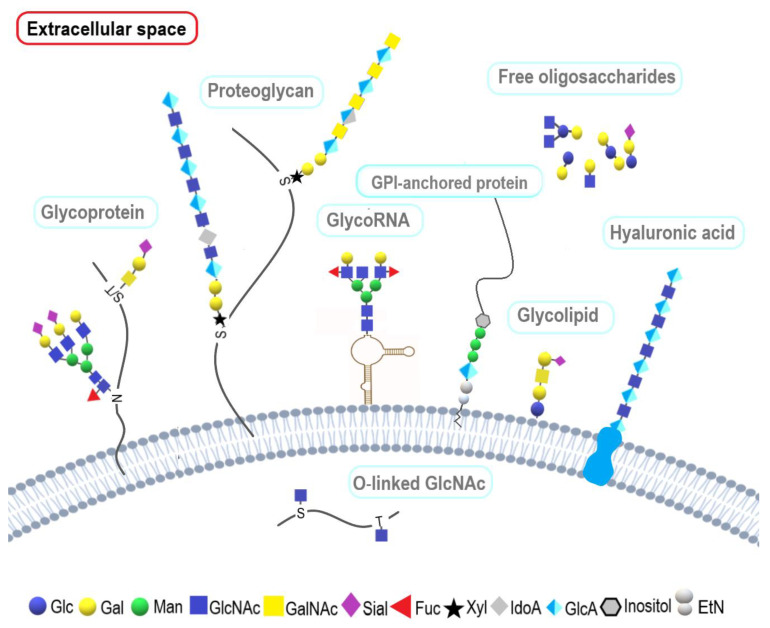
Schematic presentation of classes of glycoconjugates. Glycosylation leads to the creation of the so-called glycoconjugates, which include glycoproteins, proteoglycans, glycosphingolipids, and glycosylated RNA. In addition, glycans not covalently bound to macromolecules are present.

Glycosylation is an enzymatic process in which glycan structures are attached to proteins, lipids, and, as recently demonstrated, RNA [8]. Glycan is a general term for any monosaccharide or set of monosaccharides (oligosaccharides or polysaccharides) that are linked via covalent glycosidic bonds to another molecule. Glycosylation leads to the formation of glycoconjugates, which include glycoproteins, proteoglycans, glycosphingolipids, and glycosylated RNA (Figure 1). Additionally, glycans can exist as free oligosaccharides and non-peptide glycosaminoglycans (hyaluronic acid). For a long time, carbohydrates were considered only as a source of energy, a spare material, components of cell walls, membrane structures, mucus, and metabolites. However, ongoing research has shown that glycans play a significant role in many physiological and pathological biological processes [9]. It is also worth noting that the information about glycan synthesis is not completely written in the genome. Glycans are encoded in a complex network of at least several dozen genes that are (besides allelic variants) also affected by epigenetic and environmental factors [10].

The most crucial factors determining glycan formation are the expression of enzymes involved in their biosynthesis (i.e., glycosyltransferases and glycosidases) and the expression of genes encoding them, but also the availability of active monosaccharide donors. Consequently, glycosylation is highly species-, tissue-, and cell-specific. It depends on the current physiological/pathological state of the cell, but in some cases, there are allelic variants determining glycosylation status, e.g., glycan-based blood groups [11]. In the case of glycoproteins, we also deal with microheterogeneity (i.e., the attachment of different glycans to the same glycosylation site), which may contribute to their structural variability and affect their function (in a manner analogous to the effects of protein sequence changes caused by corresponding gene mutations) [12].

Numerous studies have shown that during neoplastic transformation, rapid changes in glycan biosynthesis result in the appearance of the so-called tumor-associated carbohydrate antigens (TACAs) on the cell surface [13]. The emergence of TACAs affects cell-cell and cell-extracellular matrix (ECM) interactions [14], survival and proliferation of cancer cells, tumor invasion, and metastasis, induction of chronic inflammation and angiogenesis, as well as immune evasion and insensitivity to apoptosis [15,16,17]. Cancer-related changes in glycan biosynthesis can result in an underexpression or overexpression of naturally occurring glycans, expression of glycans normally restricted to embryonic tissues, the appearance of incomplete or truncated structures, and, less commonly, the appearance of completely novel structures [15]. The most frequently observed changes in glycosylation in tumors and their functional significance are summarized in Table 1.

**Figure 2:**
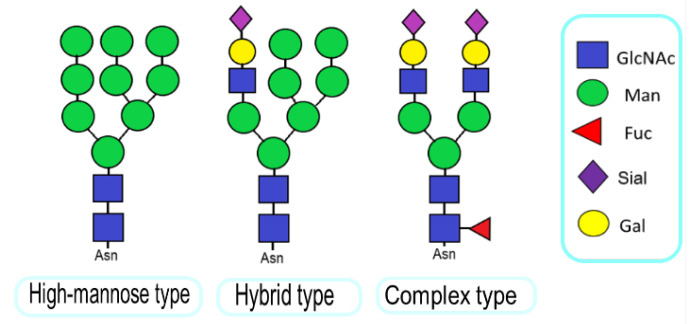
Schematic presentation of N-glycans types.

Studies on cancer-related changes in glycosylation have focused mainly on glycoproteins. Glycoproteins can be categorized into those containing N-linked glycans (nitrogen-attached), O-linked glycans (oxygen-attached), and less common C-linked glycans (carbon-attached). N-glycosylation occurs only within Asn−X−Ser/Thr amino acid sequence and, in a rare case, within Asn–X–Cys, where X can be any of the amino acids except for Pro. The key step in the N-glycan biosynthesis includes the synthesis of the precursor oligosaccharide, which is transferred en-block onto the newly synthesized peptide and is further processed into one of three types of N-glycans: high-mannose, complex, or hybrid structures (Figure 2) [18,19]. All N-glycans share a common pentasaccharide structure (Man_3_GlcNAc_2_).

In the case of O-glycosylation, the glycan is attached to the hydroxyl group of Ser or Thr residues, or to a lesser extent, to the hydroxyl group of hydroxylysine and hydroxyproline. The most common type of O-linked glycans is mucin-type O-glycans, where GalNAc is the first monosaccharide attached to Ser/Thr via α-glycosidic linkage (Figure 1). However, there are several other types of O-glycans found in glycoproteins other than mucin-type O-glycans, including β-linked O-GlcNAc, β-linked O-xylose, α-linked O-mannose, α-linked O-fucose, and also glucose and galactose, which can be both α- and β-linked. The listed monosaccharide residues attached to the above-mentioned amino acid residues by an O-glycosidic bond can then be elongated, which leads to the formation of either short chains with few residues or elongated bi-antennary structures or no elongation at all, as in the case of GlcNAcylation. Mucin-type O-glycans, unlike N-glycans, have fewer structural rules and do not share a common core structure. Subsequent elongation by successive linking of the monosaccharides to the eight types of core structures is followed by capping and sulfonation that leads to the formation of numerous different glycan structures found in mucins, i.e., glycoproteins bearing clusters of GalNAc-based O-glycans. It is worth emphasizing that the urothelium surface of the bladder wall is covered with a gel-like layer of mucins [20]. In the same layer, glycosaminoglycans can be found, which are long, linear, and highly negatively charged polysaccharides. Proteoglycans are formed through the linking of glycosaminoglycans via a special linker to the serine residue within a polypeptide chain (Figure 1). Another type of glycans found in glycoproteins is glycosylphosphatidylinositol (GPI) anchors. GPI are complex glycophospholipids covalently attached to many of the outer proteins of the eukaryote’s cell membrane (Figure 1). The function of GPI is to ensure the stable association of proteins lacking a transmembrane domain with membrane lipid rafts.

**Table 1:** Changes in glycosylation of cancer cells and their impact on cancer progression.

Change in Glycosylation	Role in Cancer	References
Increase in β1,6-branched N-glycans due to overexpression of N-acetylglucosaminyltransferase V (gnt-V)	Increased rate of metastases in mice	[19,21,22,23]
Increase in β1,4-branched tetra-antennary N-glycan due to overexpression of N-acetylglucosaminyltransferase IV (gnt-IV)	Enhanced tumor progression by lattice formation via galectin binding to poly-N-acetyllactosamines (lacnac), and the formation of sialyl-Lewis X (sle^x^)	[19,23,24]
Increase in N-glycans core fucosylation due to overexpression of α-1,6-fucosyltransferase (FUT8)	Promotion of lung cancer and melanoma progression, a critical role in antibody-dependent cellular cytotoxicity (ADCC) and immune evasion, the regulation of TGF-β, EGF, α3β1 integrin, and E-cadherin function	[19,23,25]
Increase in bisecting glcnac in N-glycans due to overexpression of N-acetylglucosaminyltransferase III (gnt-III)	Suppression of tumor progression in melanoma and mouse mammary tumors	[23,26]
Presence of Tn and T antigens and their sialylated glycoforms, sialyl-Tn (stn) and sialyl-T (ST), respectively	Interference with immune cell recognition and blocking or masking of antigenic peptides presentation by major histocompatibility complex (MHC) molecules; enhanced tumorigenic and invasive properties and promotion of immunosuppression	[23]
Increase in N-glycan α2,6 sialylation due to β-galactoside α2,6-sialyltransferase 1 (ST6GAL1) up-regulation	Increased integrin-mediated cell motility and protection from apoptosis induced by galectins, death receptor ligands, and chemotherapeutic drugs	[27]
A2,8-linked polysialic acids (polysia)	Reduced tumor cell anchorage to extracellular matrix components, making it easier for cancer cells to enter the bloodstream and interact with platelets	[14]
N-acetylglucosaminylacylation (O-glcnacylation)	Cancer risk factor, excessive nutritional intake	[28]
O-acetylated gangliosides	Protection from apoptosis	[23]
Metabolic incorporation of diet-derived N-glycolylneuraminic acid (Neu5Gc) into human glycans	Promotion of tumor growth by enhancing chronic inflammation and angiogenesis	[23]
Sialylation of Lewis^x^ and Lewis^x/a^	Crucial in extravasation	[14]
Sle^x^ and sle^a^ epitopes on glycosphingolipids	Metastatic potential in mice and tumor progression, metastatic spread, and poor prognosis of patients	[19]
Sialyl-Lewis-related structures	Influencing tumor progression by interacting with Siglecs that have immunosuppressive functions	[23]
Complete loss of glycosylphosphatidylinositol (GPI)-anchored proteins	Observed in some cases of malignant and premalignant states involving the hematopoietic system	[23]

## 3. The Role of β1,6-Branched N-Glycans in Bladder Cancer

**Figure 3:**
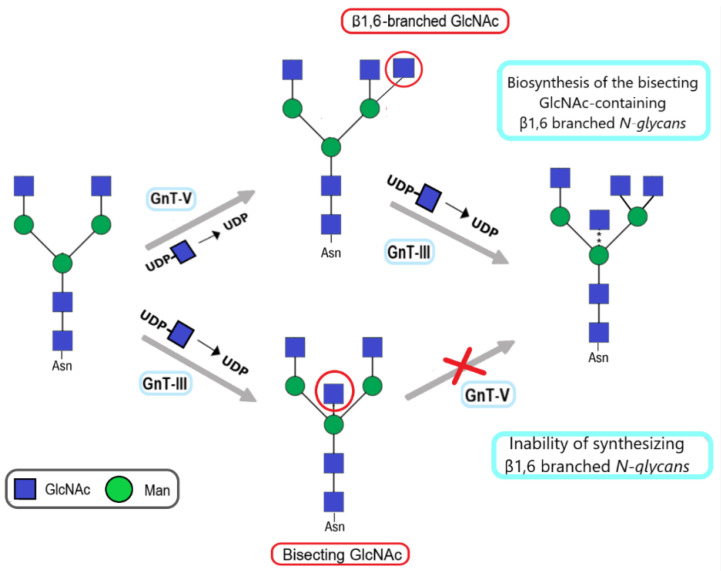
GnT-V catalyzes the formation of β1,6 branched complex type N-glycans. GnT-III catalyzes the attachment of so-called bisecting GlcNAc to complex and hybrid (not shown) type N-glycans. Glycans possessing bisecting GlcNAc are not substrates for GnT-V, which leads to the inhibition of the biosynthesis of β1,6 branched N-glycans.

The biosynthesis of β1-6 branched N-glycans relies on the activity of N-acetylglucosaminyltransferase V (EC 2.4.1.155, GnT-V, encoded by *MGAT5* gene). GnT-V catalyzes the attachment of N-acetylglucosamine (GlcNAc) residue to α1-6-linked mannose in the core structure via β1-6-linkage using UDP-GlcNAc as the donor substrate (Figure 3) [29,30]. This modification takes place in the Golgi apparatus and concerns only particular proteins, including growth factor receptors, cell adhesion molecules, enzymes, and endogenous lectins [14,31,32]. The formation of tri-/tetra antennary β1-6 branched N-glycans can modulate the half-life and stability of extracellular binding proteins, as well as their functional activity.

Furthermore, there is a link between β1,6-branched N-glycans and cancer development [15,32], and the close association between these structures and a poor prognosis of cancer patients has been reported [33,34]. Nevertheless, the clinical implications of the expression of GnT-V and β1,6-branched N-glycans vary depending on the type of cancer. So far, there are few studies related to the role of β1,6-branched N-glycans in BC. In the mid-2000s, swainsonine was used to evaluate the impact of β1,6-branched complex type N-glycans on the adhesion and migration of BC T24 cells [35]. Swainsonine is an indolizidine alkaloid, first isolated from *Swainson canescens*, which is an inhibitor of Golgi α-mannosidase II [36]. This enzyme catalyzes the removal of mannose residues from the α1-6 arm of GlcNac_2_Man_5_GlcNAc, and its inhibition leads to the accumulation of high mannose (Man_4_GlcNAc_2_ and Man_5_GlcNAc_2_) and hybrid-type N-glycans in glycoproteins. It was shown that after swainsonine treatment, BC T24 cells significantly increased their adhesion to collagen IV, laminin, and fibronectin. Swainsonine treatment also diminished the migration rate in wound healing assay, indicating the role of β1,6-branched complex type N-glycans in this process [35]. Additionally, non-malignant urothelial HCV-29 cells displayed higher adhesiveness and weaker migratory properties than T-24 BC cells [36,37]. It can be partly attributed to the different glycosylation of α3β1 integrin. Only BC cells showed the presence of β1,6-branched N-glycans on both α3 and β1 integrin subunits [38,39], whereas the level of the α3 integrin subunit expression in both cell lines showed no statistically significant differences [40]. Interestingly, in the case of cadherins, the presence of β1,6-branched N-glycans was demonstrated only for T24 cells but not for HCV29 cells [41].

Although in vitro research suggests that β1,6-branched N-glycans promote BC progression, studies using patient tissues showed that GnT-V expression is lower in advanced stages of BC, while higher GnT-V expression correlates with longer disease-free survival [42,43]. Nevertheless, GnT-V activity in BC tissues was shown to be slightly elevated in comparison to non-malignant specimens [44]. Recently, a link between the activity of GnT-V and response to chemotherapeutic gemcitabine in BC was studied [45,46]. Gemcitabine treatment of T24 cells increased the expression of GnT-V and the amount of β1,6-branched N-glycans on human equilibrative nucleoside transporter 1 (hENT1). On the other hand, GnT-V silencing in T24 cells caused a significant decrease in the amount of β1,6-branched N-glycans on hENT1. It led to decreased uptake of gemcitabine by BC cells, inhibition of drug-induced apoptosis (due to reduced expression of active caspases 3, 8, and 9), as well as G2/M cell cycle arrest. Thus, the expression of β,1-6 branched N-glycans, and GnT-V is related to low malignant potential and good prognosis for BC patients.

## 4. The Role of Bisecting N-Acetylglucosamine in Bladder Cancer

N-acetylglucosaminyltransferase III (EC 2.4.1.144, GnT-III, encoded by *MGAT3* gene) catalyzes the transfer of N-acetylglucosamine (GlcNAc) residue from UDP-GlcNAc to core mannose of N-glycan with a β1,4 linkage forming a bisecting GlcNAc structure (Figure 3) [47]. The introduction of a bisecting GlcNAc residue causes a conformational change in the glycan and prevents further processing and the biosynthesis of β1,6-branched N-glycans catalyzed by GnT-V [24]. In the context of a tumor, GnT-III and bisecting GlcNAc are mostly considered suppressors of tumor growth, epithelial-mesenchymal transition, and metastasis [24,48]. To our knowledge, there are only two papers describing the role of bisecting GlcNAc in bladder cancer.

First, it has been shown that there is a link between the increase in bisecting GlcNAc on fibronectin from patients’ urine and the pathological stage and grade of BC [44]. The binding affinity of fibronectin to wheat germ agglutinin (WGA) was, on average, 3.26 times higher for BC samples compared to healthy controls. Additionally, the GnT-III activity was, on average, 34 times higher in BC samples, and its activity positively correlated with the stage and grade of BC. These observations regarding the elevated level of bisecting GlcNAc in BC have recently been confirmed by another study on N-glycosylation of serum immunoglobulins (Igs) in urothelial carcinomas (UC), most of which originate from bladder [49]. The results of that study clearly showed that bisecting GlcNAc on Igs was significantly accumulated in UC patients. Summing up, even though the number of studies on the role of bisecting GlcNAc in BC is limited, they contradict the hypothesis that this modification has tumor-suppressive functions.

## 5. The Role of O-GlcNAcylation in Bladder Cancer

**Figure 4:**
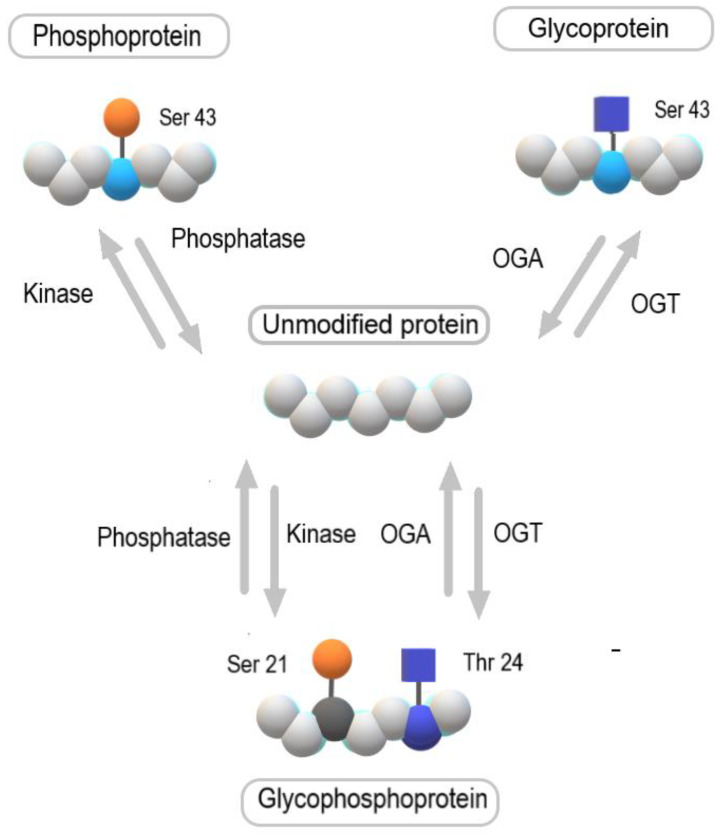
The crosstalk between O-GlcNAcylation and phosphorylation of serine and threonine residues, and the antagonistic relation between OGT-kinase and OGA-phosphatase.

O-GlcNAcylation is a reversible and dynamic post-translational modification of cytoplasmic, nuclear, mitochondrial, and plasma membrane proteins involving the attachment of a single GlcNAc residue to serine or threonine residues. O-GlcNAcylation modulates the function of proteins involved in fundamental cellular processes such as transcription, translation, signal transduction, metabolism, cytoskeletal functions, and cell division [50,51,52]. The process of O-GlcNAc attachment is catalyzed by O-GlcNAc transferase (EC 2.4.1.255, OGT, encoded by *Ogt* gene), whereas the removal of the attached GlcNAc residue is catalyzed by O-GlcNAcase (EC 3.2.1.169, OGA, encoded by *MGEA5* gene) (Figure 4) [53]. The level of O-GlcNAcylated proteins is regulated by the nutritional state of the cell as well as metabolic, oxidative, and proteotoxic stress. Finally, O-GlcNAcylation directly or indirectly competes with phosphorylation for the same serine/threonine residues. It provides complex cross-communication between the two posttranslational modifications for control of the function of different proteins (Figure 4) [51,54].

Furthermore, O-GlcNAcylation is highly responsive to environmental nutrient concentrations, i.e., glucose and glutamine [55]. Numerous epidemiological studies have shown that obesity and diabetic conditions significantly increase cancer risk and enhance O-GlcNAcylation. [28,56,57,58]. Increased O-GlcNAcylation and changes in OGT and/or OGA expression have been demonstrated in several types of cancer. Moreover, O-GlcNAcylation participates in the regulation of the epigenome and transcription factors activity (MYC, p53, NFκΒ, β-catenin, FOXM1, estrogen receptor (1) as well as in reprogramming of the metabolic network of cancer cells to promote tumor growth. O-GlcNAcylation can also alter cancer cell proliferation by (2) modulating PI3K/Akt activity and participating during the cell cycle at various stages, (3) promoting invasiveness and metastasis by affecting the E-cadherin/β-catenin system, (4) and/or by promoting the expression of metalloproteinases. Finally, O-GlcNAcylation is involved in inducing immune surveillance in the tumor microenvironment and is involved in the activation and differentiation of T lymphocytes and macrophages.

To our knowledge, the first papers on the role of O-GlcNAcylation in BC were published over 20 years ago. It was shown that OGT mRNA was expressed in the urine of 51.7% of BC patients but not in the urine of healthy individuals. Moreover, the OGT mRNA level was significantly higher in the urine of patients with grades II and III of BC in comparison to grade I [59]. Consistent with this observation, other studies demonstrated the tumor-promoting activity of GlcNAcylation in BC [60,61,62]. Evaluation of OGT expression and GlcNAcylation levels by immunohistochemistry, the sequencing data, and Western blotting showed their upregulation in BC tissues and cells vs. normal tissues/cells [60,61,62]. Importantly, OGT levels were higher in MIBC than in NMIBC. On the other hand, OGA expression was found to be lower in cancer tissues [61].

The direct involvement of GlcNAcylation in promoting BC cell proliferation in vitro and tumor xenograft growth in vivo, as well as in inhibiting apoptosis and autophagy, was also proven by OGT knockdown studies [60,61]. In vitro, O-GlcNAcylation was found to promote migration and invasion of BC cells and to induce cell cycle arrest [61]. It was later proven that autophagy in BC cells could be induced by blocking of O-GlcNAcylation of the autophagy suppressor AMP-activated protein kinase (AMPK) [63]. On the other hand, the inhibition of apoptosis in BC cells may result from cyclin-dependent-like kinase 5 (CDK5) modification by O-linked GlcNAc [62]. Summing up, the removal of O-GlcNAc or inhibition of O-GlcNAcylation may help to induce autophagy in BC cells or prevent BC cells from escaping apoptosis.

Furthermore, it has been shown that hyper-O-GlcNAcylation due to nucleolar and spindle-associated protein 1 (NUSAP1) overexpression might promote BC progression [64]. NUSAP1 is a microtubule-binding protein located mainly in the nucleolus, the up-regulation of which enhances cancer cell invasive and migratory potential and is associated with patients’ poor survival. Another study demonstrated that a reduction of OGT expression increases the sensitivity of bladder cancer cells to cisplatin [60]. Moreover, the acquired resistance to gemcitabine and paclitaxel was shown to induce the overexpression of OGT in BC [65]. It suggests the pathological role of OGT in the development of multidrug resistance in BC.

## 6. The Role of Total Sialylation in Bladder Cancer

Sialylation is an enzymatic process during which sialic acid residues are covalently attached through α2,3, α2,6, and α2,8 binding to the terminal positions of glycan chains on various acceptors, such as glycoproteins, glycolipids, polysaccharides, including polysialic acid chains. Sialic acids (Sia) represent a family of more than 50 derivatives of neuraminic acid, a nine-carbon monosaccharide consisting of a six-membered carbon ring (C1–C6) with a three-carbon C7–C9 glycerol chain attached. The main derivatives of neuraminic acid are N-acetyl-neuraminic acid (Neu5Ac) and N-glycolyl-neuraminic acid (Neu5Gc), which are its N- and O-substituted derivatives, respectively. Neu5Ac and Neu5Gc can be further modified by the addition of O-acetyl, O-methyl, sulfate, O-lactyl, and phosphate groups at various positions. Importantly, NeuGc is selectively expressed in cancer cells, whereas it is absent or present in low amounts in normal tissues because the human *CMAH* gene is irreversibly mutated. CMP-Sia is a donor of sialic acids in the sialylation reaction that is catalyzed by members of the sialyltransferase family, which in humans consists of 20 subtypes [66,67].

It is known that cancer cells often exhibit elevated levels of sialylated glycoconjugates. This overexpression may result from the deregulation of the enzymatic activity of sialyltransferases or neuraminidases (glycosidic hydrolases that catalyze the removal of sialic acid residues from glycoconjugates) or both [66,68,69]. Abnormal sialylation has been found to affect the adhesive, migratory and invasive properties of tumor cells, epithelial-mesenchymal transition (EMT), metastasis formation, signal transduction, angiogenesis, as well as immune system evasion, avoidance of apoptosis and cell death, and reduced efficacy of chemotherapy and radiotherapy [70,71,72,73,74].

Total sialic acid (TSA) has been widely recognized as a specific tumor marker and potential therapeutic target due to its higher expression on the surface of cancer cells. In BC, the enzyme responsible for the increase of TSA is ST3GAL6, an enzyme that has α-2,3-sialyltransferase activity toward the Gal-β1,4-GlcNAc structure on glycoproteins and glycolipids [75]. ST3GAL6 has been shown to be downregulated by the transcription factor GATA3, which is one of the key factors maintaining luminal differentiation of urothelial cells [76]. On the other hand, overexpression of ST3GAL6 increased the migration and invasive capacity of BC cells and correlated with the poor prognosis of bladder cancer patients [75]. Elevated serum sialic acid levels have also been correlated with the extent of malignancy, and they appear as a promising biomarker of urothelial tumors [77].

## 7. The Role of Mucin-Type O-Glycans in Bladder Cancer

**Figure 5:**
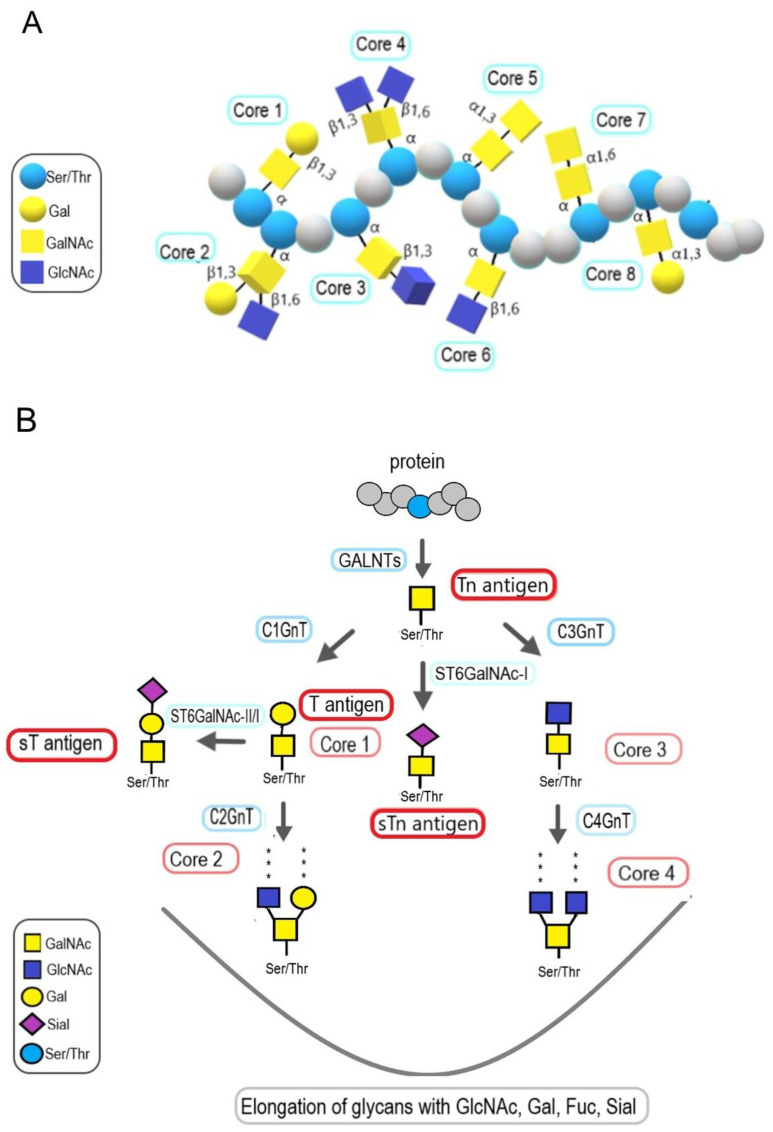
Mucin-type O-glycosylation is initiated in the Golgi apparatus by polypeptide N-acetylgalactosaminyltransferases (GalNAc-Ts, twenty isoforms), which initiates the action of numerous glycosyltransferases catalyzing the attachment of galactose, GlcNAc, fucose, and sialic acid residues that result in the extension of GalNAc into numerous different structures. Mucin-type O-glycosylation leads to the synthesis of eight different core structures. (**A**) Core structures of mucin-type O-glycans. (**B**) Biosynthesis of O-glycan core 1–4 structures, their sialylated forms, and enzymes taking part in these processes. During the neoplastic transformation, shortened O-glycan chains are formed. The most common forms are the Tn and T antigens and their sialylated forms.

Another type of glycans that are covalently attached to serine and threonine residues of proteins via O-glycosylic linkage are mucin-type O-glycans. Mucin-type O-glycosylation, which is the dominant type of O-glycosylation, is an evolutionarily conserved post-translational modification in animals. Mucin-type O-glycosylation is initiated in the Golgi apparatus by a family of 20 homologous polypeptide N-acetylgalactosaminyltransferases (EC 2.4.1.41, GalNAc-Ts). They catalyze the first step of biosynthesis by forming GalNAcα1-O Ser/Thr linkage in O-glycoproteins, known as Tn antigen [78,79]. Importantly, GalNAc-T1 is necessary for the self-renewal and tumor-initiating capacity of BC stem cells [80]. The O-GalNAc residues are further processed by the addition of galactose and GlcNAc residues, leading to the synthesis of 8 types of core structures. Core structures are further elongated by the addition of galactose, GlcNAc, fucose, and sialic acid residues (Figure 5). Mucin-type O-glycans are found on cell surface proteins, i.e., mucins and other glycoproteins. Mucins are involved in the protection of epithelial cell surface as well as in the sorting and secretion of glycoproteins, cell adhesion, migration, trafficking of hematopoietic cells, host-pathogen interaction, and immune responses [79,81,82,83,84,85]. Importantly, altered expression of these structures is known to be associated with several diseases, including cancer.

A number of excellent reviews have been published in recent years that describe the role of mucins in cancer, to cite some of them [86,87,88,89,90,91,92,93,94,95,96,97,98,99,100]. Changes in the expression, distribution, and glycosylation of mucins have been well documented in several cancers, providing a rationale for exploring their use as both diagnostic and prognostic markers, as well as targets for the development of cancer therapies. The role of mucins in cancer pathogenesis stems from the effects they exert on numerous signaling pathways, such as NF-kB, ERα, HIF, MAPK, p53, c-Src, Wnt, and JAK-STAT, which results in the reprogramming of cancer-related genes expression profiles. Numerous studies have confirmed that mucins are involved in the regulation of cancer cell proliferation, growth, migratory and invasive properties, their detachment and reattachment during metastasis, self-renewal of cancer stem cells (CSCs), avoidance of apoptosis, escape from immune surveillance, drug metabolism, and induction of drug resistance. Moreover, glycans in mucins serve as ligands for various carbohydrate-binding proteins such as galectins, selectins, and siglectins, thus changes in their structure are responsible for altering the interaction of cancer cells with other cells, e.g., lymphocytes, platelets, and endothelial cells.

### 7.1. The Role of Mucin Expression in Bladder Cancer

As mentioned above, mucins (MUCs) are the main carriers of mucin-type O-glycans. MUCs are high molecular weight glycosylated proteins present in the epithelial lining of certain tissues and ducts. Above twenty types of mucins have been identified. They have been divided into two major groups, i.e., membrane-bound and secreted. It is well known that abnormal expression and glycosylation of mucins contribute to tumor survival and proliferation in many types of cancers [89]. Regarding BC, MUC1, MUC2, MUC4, MUC6, and MUC7 have been shown to be expressed by urothelial cells [101,102].

It was found that the differences in the distribution of mucins within the epithelium of the urinary tract as well as in blood and urine samples have diagnostic and prognostic potential. MUC-1 expression has been found to increase when primary BC acquires a more aggressive phenotype, and its high expression is correlated with higher tumor grade and worse prognosis [103,104,105,106,107]. MUC2 and MUC6 expression was associated with low-grade tumors and presented an inverse correlation with cancer-specific deaths [105]. On the other hand, MUC4 expression was linked to cancer-specific deaths [104,105]. Finally, MUC7 expression was detected only in samples of invasive transitional cell carcinomas [108,109,110,111].

Another member of the mucin family proteins, MUC16, also known as cancer antigen 125 (CA125), has been associated with shorter disease-free periods and poorer overall survival rates of BC patients [112,113]. MUC16 expression was also associated with the immunosuppressive tumor microenvironment and gemcitabine/cisplatin resistance [112,113]. Furthermore, CD164, another member of this family involved in tumor maintenance and progression, has been associated with poor clinical outcomes of BC patients [114]. Last but not least, Cluster of Differentiation 44 (CD44), the major surface receptor for hyaluronic acid, is another glycoprotein with mucin-type O-linked glycans. CD44 is a multifunctional protein involved in cell adhesion, cell-matrix adhesion, cell migration, leukocyte homing and activation, tumor invasion, and metastasis. CD44 expression has been positively correlated with tumor aggressiveness and the response of bladder cancer to radiation, as well as associated with drug-resistant phenotype and poor prognosis [115,116,117,118]. It has also been proven that CD44 O-glycosylation status regulates cancer aggressiveness [119].

Finally, MUC1 (also known as CA15-3), whose expression correlates with high BC grade, takes part in epithelial surface protection, regulation of receptor kinase signaling, and cell adhesion by interacting with β-catenin and cyclin D1. In cancer, overexpression of MUC1 interferes with cell adhesion, protects tumor cells from immune recognition, and promotes metastasis. Furthermore, MUC1, an autoantigen, is altered during the malignant process and induces immune responses. Recently, MUC1 has been shown to play a key role in the acquisition of drug resistance [99,120] and metabolic reprogramming leading to increased glycolysis, glucose uptake, and lactate production in BC [99]. Interestingly, high expression of MUC1 was associated with a favorable prognosis for patients with BC when the expression of the epidermal growth factor (EGF) receptor HER3 was also high [121,122]. MUC1 is highly O-glycosylated on Ser/Thr residues and carries core 2 mucin-type O-glycans, whose role in bladder cancer is described in Section 7.3.

Summing up, not only changes in the expression of mucins but also in their post-translational modifications are a common feature of many tumors originating from human epithelial cells. Mucin-type O-glycans associated with malignancies are incompletely synthesized O-linked glycans. Their presence, together with the accumulation of precursors, results in the loss of the normal O-glycan antigens.

### 7.2. The Role of Tn and T Antigens in Bladder Cancer

Tn antigen, T antigen, and their sialic acid counterparts, i.e., the sialyl-Tn (STn) antigen and the (mono-/di-) sialyl-T (ST) antigen (Figure 5), are among the most intensively investigated TACAs in BC. Moreover, in BC tissues, a predominance of sialoglycans over neutral glycoforms of Tn and T antigens has been observed [112]. Tn and STn antigens are not found in normal urothelium because Tn is masked by covalently bounded terminal carbohydrate residues and is, therefore, hardly expressed [123]. The premature stop of Tn antigen elongation by its sialylation is observed in tumors and leads to the synthesis of STn antigen. STn antigen is the most widely studied glycotype in cancer, and its biosynthesis is controlled by the ST6 N-acetylgalactosaminide α-2,6-sialyltransferase 1 (ST6GALNAC-I), which catalyzes the transfer of NeuNAc to the O-6 position of GalNAc residues linked to Ser/Thr. Increased ST6GALNAC-I activity has been associated with the efficacy of treatment with Bacillus Calmette-Guérin (BCG), the gold standard adjuvant immunotherapy for NMIBC after transurethral resection, to which one-third of patients fail to respond. The analysis of BC tissues showed that patients with elevated activity of ST6GALNAC-I, along with high expression of interleukin-6 mRNA, responded to BCG treatment [124]. In addition, STn antigen expression alone or in combination with S6T antigen, i.e., T antigen isotype having α2,6-linked sialic acid residue, has been associated with lower recurrence rates after BCG treatment [125]. Moreover, STn antigen-expressing cancer cells internalized more BCG and showed higher rates of BCG-induced apoptosis compared to STn-negative cancer cells [125].

In BC, the STn antigen has been associated with high-grade tumors, invasion of the muscle layer, and overall poor survival [112,123,125,126,127]. In addition, a strong link between STn antigen expression, tumor dissemination, and metastasis was stated based on high STn antigen expression in circulating tumor cells (CTCs) [128,129]. Moreover, STn antigen has been shown to induce morphological changes in BC cells, modulate their cell-cell and cell-matrix adhesion properties, increase invasive capacity, and enable immune escape [123,125,127,130]. In the immunological context, STn antigen is known to contribute to evasion from immune surveillance. For instance, STn antigen-bearing mucins inhibit the cytotoxicity of natural killer (NK) cells, thereby impairing their function [131]. Glycoproteomic analyses of BC tissues have also revealed that STn antigen was carried by 143 glycoproteins involved in mediating protein binding, cell-cell communication, cell signaling, regulation of metabolic processes, and hydrolase catalytic activities [112]. These observations regarding STn antigen-bearing glycoproteins were in line with previous in vitro studies [127]. Among STn antigen-expressing proteins, several integrins and cadherins, as well as CD44, MUC1, and MUC16, were identified [112,127]. Interestingly, overexpression of STn antigen has been shown to modulate CD44-mediated invasion and regulate oncogenic pathways governed by CD44 [118]. Moreover, there is a positive correlation between STn antigen expression and the higher proliferation index of BC cells based on p53 and Ki-67 biomarkers [132]. Finally, hypoxia, which is typical for advanced-stage bladder tumors [133,134], may promote STn antigen overexpression in BC cells [127]. In contrast, glucose deprivation has led to the expression of more Tn antigens and, to less extent, STn antigens [135]. HOMER3 has been identified as one of the main glycoproteins triggered by hypoxia and glucose deprivation, whose expression in bladder cancer patients has been associated with more aggressive primary tumors and distant metastases [136].

STn antigen expression is clinically important, not only as a diagnostic and prognostic marker in cancer (CA72-4 serological test) but also as a target of therapeutic strategies. STn antigen-based vaccines have had limited success, probably because STn antigen-expressing cancer cells impair dendritic cell (DC) maturation, downregulate the expression of proinflammatory cytokines (IL-12 and TNF-α), and endow DCs with a tolerogenic function [130]. In addition, STn antigen interactions with Siglec-15 present on macrophages have been shown to enhance the secretion of TGF-β, a key regulator of immune tolerance [137]. However, a novel CAR T cell-based treatment strategy targeting STn antigen has recently been developed in mice. The treatment effectively promoted the secretion of proinflammatory cytokines and tumor cell lysis in vitro and in experimental mice [138]. STn antigen has also been selected as a surrogate marker of BC associated with *Schistosoma haematobium* infection [139].

Furthermore, core 1 β1,3-galactosyltransferase (C1GnT) is the enzyme catalyzing the conversion of Tn antigen to T antigen, also known as core 1 structure. C1GnT has been shown to modulate the expression of target glycoproteins, such as MUC1 in spontaneous gastritis and gastric cancer [140] and MUC16 in pancreatic adenocarcinomas [141]. On the other hand, the loss of C1GnT resulted in Tn antigen enrichment on CD44 in pancreatic cancer [142]. In BC tissues, the expression of C1GnT and T antigens was found to be higher than in noncancerous bladder tissues [143]. In addition, silencing of C1GnT inhibited the migration and proliferation of BC cells by modifying target glycoproteins, including MUC16. It confirmed the pro-metastatic and proliferative function of upregulated C1GnT. Furthermore, C1GnT expression depends on the co-expression of its specific molecular chaperone, i.e., COSMC protein. COSMC, localized in the endoplasmic reticulum, ensures proper folding and prevents aggregation and proteasomal degradation of C1GnT. COSMC can also interact with partly denatured C1GnT and partially restore its activity. Knock out, silencing, or mutations in the COSMC gene disturb the balance of O-GlcNAcylation, and such abnormal levels of O-glycosylation are closely associated with tumor growth and differentiation [144,145]. 

The T antigen is also the precursor of the ABO blood group determinants. In BC patients, the loss of ABO(H) blood group determinant expression is associated with advanced forms of the disease and an unfavorable prognosis [146]. The overexpression of T antigen and its sialylated form (ST antigen) has also been associated with poor prognosis [147]. T antigen is a substrate for a few sialyltransferases. However, upregulation of ST3Gal-I has been shown to be one of the main mechanisms responsible for T antigen sialylation, and much higher mRNA levels of ST3Gal I were observed in malignant BC [147]. Interestingly, ST3Gal-I overexpression and the consequent replacement of T antigen with ST antigen having α2,3-linked sialic acid have been shown to induce transcriptomic changes in BC cells. These changes included the decreased expression of several genes involved in different mechanisms of DNA repair, the accuracy of chromosomal segregation, increased susceptibility to oxidative damage, and a stronger inflammatory response of macrophages [148]. Thus, ST antigen overexpression mediated by STGAL-I upregulation appears in the initial stages of oncogenic transformation and persists into more advanced stages of BC [112]. Both whole serum mono- and di- sialylated T antigens were increased in BC patients [149]. Finally, glycoproteomic analysis of two BC cell lines (T24 and 5637 cells) has revealed that over 90% of identified proteins potentially expressed ST antigen [135].

### 7.3. The Role of Core 2 Structures in Bladder Cancer

In addition to sialylation, T antigen can be modified to form core 2 mucin-type O-glycans that provide a scaffold for subsequent poly-N-acetyllactosamine synthesis. The conversion of T antigen to core 2 is catalyzed by core 2 β-1,6-N-acetylglucosaminyltransferase (C2GnT) (Figure 5), the expression of which positively correlates with both BC stage and grade [150]. Moreover, it has been found that BC patients with C2GnT expression had significantly shorter survival rates than patients not expressing C2GnT [151].

Upregulation of C2GnT has been shown to be a mechanism by which BC cells evade NK immunity. NK cells are key antitumor-effective cells that detect and eliminate circulating cancer cells at the early stages of the disease. One of the substrates of C2GnT is MHC class I-related chain A (MICA), a ligand expressed by cancer cells for an NK-activating receptor (NKG2D). Modification of MICA with poly-N-acetyllactosamine reduces its affinity for NKG2D on NK cells, which allows cancer cells to evade NK-mediated cell death [151]. Another mechanism that allows evading NK response is also related to the presence of mucin core 2 with poly-N-acetyllactosamine on MUC1, which enables the binding of MUC1 through to galectin 3 [150]. It interferes with the access of the tumor necrosis factor-related apoptosis-inducing ligand (on NK cells) to the surface of cancer cells, thereby prolonging the life of CTCs. Furthermore, it has been shown that bladder cancer cells expressing C2GnT have been highly susceptible to cytotoxic T lymphocyte (CTL)-mediated response. It is possible due to the presence of O-glycans of mucin core 2 with poly-N-acetyllactosamine on human leukocyte antigen class I (HLA I), which, due to binding with galectin 3, ensures retention of HLA I on the cell-surface [152]. Thus, evasion of CTL-mediated antitumor immunity is associated with the loss of core 2 structures on human leukocyte antigen HLA I as downregulation of C2GnT in metastatic cells has been reported [152].

It has also been found that the total mRNA level of β1,4-galactosyltransferase-1 (β4GalT1), an enzyme that catalyzes the transfer of galactose from UDP-Gal to GlcNAc and forms the poly-N-acetyllactosamine structure, was significantly higher in BC tissues than in normal ones [153]. Moreover, the level of upregulated expression of a long-form of β4GalT1 (β4GalT1-L) was higher than that of short-form β4GalT1 (β4GalT1-S) in BC cells. These forms were also differently localized in cells and played different roles in drug resistance. Chemotherapy upregulated the expression of β4GalT1-S. On the other hand, the expression of β4GalT1-L was downregulated and resulted in the decreased amount of poly-N-acetyllactosamine structure on mouse double minute 2 homolog (MDM2), a key negative regulator of p53 because MDM2 is preferentially glycosylated by β4GalT1-L. Therefore, chemotherapy-induced overexpression of β4GalT1-S may result in the acquisition of drug resistance and cell stemness through the decrease in poly-N-acetyllactosamine on MDM2, followed by upregulation of p53 and its decreased ubiquitination.

## 8. Fucosylation in Bladder Cancer

L-Fucose (6-deoxy-L-galactose) is another sugar residue found in glycans in all mammalian cells. Fucose is transferred onto the glycan chains from GDP-fucose, and the process is catalyzed by several fucosyltransferases (FUTs) [154]. So far, 13 genes encoding FUTs have been identified in the human genome. The majority of FUTs from the Golgi apparatus can modify N-linked glycans, whereas O-fucosyltransferases are mostly localized in the endoplasmic reticulum [155]. Based on the location of fucose residue within the glycan chain, fucosylation is divided into core fucosylation (α-1,6-linked fucose), whose formation is catalyzed only by FUT8, and terminal fucosylation (α-1,2- or α-1, 3/4 -linked fucose), whose formation is catalyzed by FUT1-FUT4, FUT6, FUT7, and FUT9 [154]. Regarding the biological role of fucosylated glycans, ABO blood antigens are the most common fucose-containing glycans. It has also been demonstrated that fucosylated glycans are crucial for selectin-mediated leukocyte extravasation, lymphocyte homing, and pathogen-host interactions. Fucosylated proteins are also widely involved in signal transduction.

Changes in fucosylation pattern may be due to the altered availability of GDP-fucose, a monosaccharide donor, or abnormal expression of FUTs and/or α-fucosidase, which may affect the proliferation and survival of cancer cells. Overexpression of α-1,6-fucosyltransferase has been found to result in increased attachment of fucose residue to the innermost GlcNAc in N-glycans core structure, and such a change is a major feature of hepatocarcinogenesis in humans [156]. Aberrant fucosylation has also been described in glioblastoma as well as in breast, colorectal, lung, and prostate cancers [157]. BC cells have been shown to possess a high expression of core fucosylated complex type N-glycans and a low expression of terminally fucosylated complex type N-glycans [158]. Importantly, elevated levels of plasma glycoproteins with terminally fucosylated glycans can distinguish BC patients from healthy controls [159].

Data available in the literature have clearly indicated the involvement of fucosylation in the formation of metastasis sites in BC. N-glycan expression profiling in transforming growth factor-beta (TGFβ)-induced EMT using normal ureter epithelial HCV-29 cells showed decreased expression of α-L-fucosidase, which contributed to increased expression of fucosylated N-glycans [160]. Similarly, a comparison of N-glycan profiles of TGFβ-treated vs. control BC cells by MALDI-TOF/TOF-MS revealed increased fucosylation in TGFβ-treated cells. These findings were consistent with the results of the lectin microarray analysis. Fucα1-2Galβ1-4GlcNAc structure, which was recognized by *Ulex europaeus* agglutinin-I (UEA-I lectin), was approximately two-fold higher in TGFβ-treated cells than in control BC cells [160].

Furthermore, calreticulin (CRT) has been demonstrated to affect cell adhesion and metastasis of BC cells. CRT stabilized mRNA for FUT-1 and thus regulated *FUT*-*1* gene expression. In the same study, CRT indirectly affected β1 integrin-mediated cell adhesion by altering the level of α1,2-linked fucosylation (FUT-1-dependant) of the β1 integrin subunit. The study also indicated that α1,2-fucosylation of the β1 integrin subunit promoted its activation instead of modifying the integrin-binding sites [161]. Another group of cell adhesion molecules involved in BC progression is cadherins. Positive reaction with *Aleuria aurantia agglutinin* (AAA) has confirmed the presence of fucose residues on N-cadherin from highly invasive T24 BC cell line and their absence in the case of three less invasive cell lines [41]. Since AAA specifically recognizes not only α1,6-linked “core” fucose residue and a Fucα1,2Galβ1,4GlcNAc sequence (blood group H(O) determinant) but also a Galβ1,4(Fucα1,3)GlcNAc sequence (Lewis^X^ determinant), N-cadherin from T24 cells might possess both core fucosylated and/or α1,2/3-fucosylated glycans.

Furthermore, FUT7 expression in BC has been shown to correlate positively with the number of tumor-infiltrating lymphocytes, tumor growth and invasiveness, cancer cell migration, and the occurrence of EMT [162]. Moreover, in another study, BC cell lines derived from invasive tumors have been demonstrated to display increased expression of FUT6 and FUT7, whereas cell lines from non-invasive tumors or normal bladder epithelia have been negative for FUT6 and FUT7 expression [163]. These results suggest that elevated levels of FUT6 and FUT7 should be evaluated as potential prognostic biomarkers in BC.

Another potential and specific diagnostic target is the glycoform of α3 integrin subunit (ITGA3) from the urine of BC patients. Detection of fucosylated ITGA3 in the ITGA3-UEA assay (anti-ITGA3 antibody and fucose-specific *Ulex europaeus* agglutinin (UEA lectin) conjugated on nanoparticles) has shown that this recognized glycovariant significantly discriminate BC patients from benign hyperplasia patients [164]. Such an assay has also been performed using extracellular vesicles (EVs) derived from BC patients’ urine and BC cell-conditioned media [165]. This study showed that a biomarker combination consisting of ITGA3 and fucose is highly expressed on EVs derived from BC cells and BC urine samples and holds promise in the detection of urological pathologies [164]. More information regarding the glycosylation of EVs in BC is provided in Section 10.

Last but not least, α-1-acid glycoprotein (AGP) is a highly heterogeneous protein that can acquire different glycosylation statuses. With the use of capillary zone electrophoresis coupled with tandem mass spectrometry (CZE-MS), it has been shown that samples from BC patients vs. healthy controls display a higher abundance of AGP isoforms containing tri- and tetra-antennary fucosylated N-glycans [166]. This suggests that fucosylated AGP should also be considered as a potential biomarker of BC.

Finally, fucose residues are also present in a very specific group of glycan epitopes called Lewis antigens. The role of fucosylated Lewis antigens in BC will be discussed in detail in Section 9.

## 9. Lewis Antigens in Bladder Cancer

**Figure 6:**
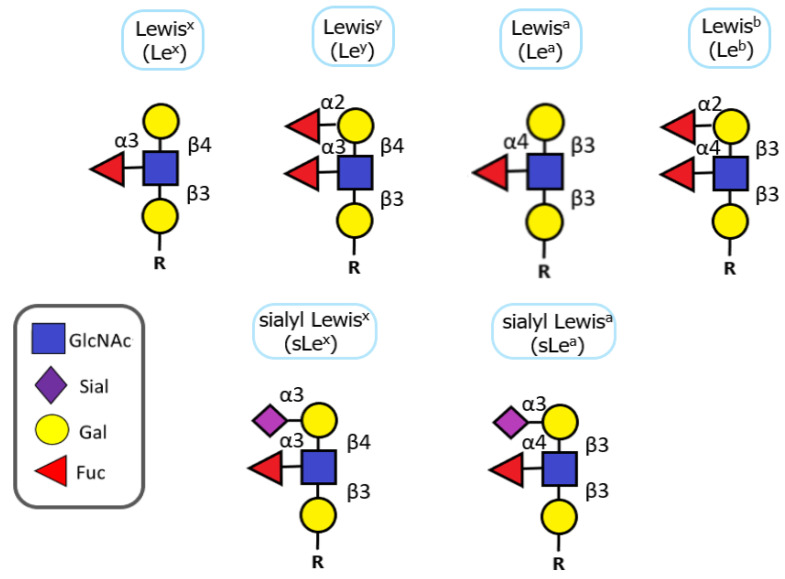
Types of Lewis (Le) antigens and their sialylated forms. Le antigens are terminal fucosylated carbohydrates present on the cell surface, glycoproteins, or glycolipids. Lewis antigens are covalently attached to the protein by binding to asparagine (Asn) or serine and threonine (Ser/Thr) residues. The presence of Lewis antigens is dependent on the expression of fucosyltransferase (FUT).

The Lewis antigen system is a human blood group system consisting of type I and type II Lewis antigens, which are terminally fucosylated glycan epitopes. The difference between type I and type II Lewis antigens is the type of glycosidic bond present in their structure, i.e., Galβ1-3GlcNAc in type I versus Galβ1-4GlcNAc in type II Lewis antigens. All Lewis antigens (H1, H2, Lewis^a^ (Le^a^), Lewis^b^ (Le^b^), Lewis^x^ (Le^x^), and Lewis^y^ (Le^y^)) contain the same three sugar residues (GlcNAc, Gal, and Fuc). Further elongation with sialic acid creates more complex glycan structures, e.g., sialyl Lewis^a^ (SLe^a^) or sialyl Lewis^x^ (SLe^x^) (Figure 6). All fucosyltransferases involved in the synthesis of Lewis antigens (FUT1–7 and FUT9) possess a unique substrate specificity that contributes to the high complexity and bioavailability of Lewis antigens in naturally occurring glycoconjugates [165]. Overexpression of mentioned fucosyltransferases and Lewis antigens has already been described in many types of cancer, including BC [167].

Neo-expression of the Le^x^ antigen, which is absent in normal urothelium, has been noted in over 85% of urothelial carcinoma regardless of tumor stage and grade [168]. In addition, immunocytological detection of the Le^x^ antigen on exfoliated bladder cells has improved the efficiency of BC detection, particularly of low-grade and low-stage neoplasms [169]. Currently, the Le^x^ antigen is considered a marker of malignant transformation in BC, and its expression has been positively correlated with the stage, grade, and metastatic potential of a tumor [167]. Moreover, the decreased expression of the Le^x^ antigen on human neutrophils has been associated with better prognosis in patients after bladder resection [170].

Furthermore, a recent report on cell lines from various stages of BC has shown that low-grade BC cell lines expressed elevated levels of the fucosylated Le^x^ antigen, while normal bladder epithelial cells lack the expression of this antigen [171]. T24 (grade 3) and TCCSUP (grade 3) cells also lack the Le^x^ antigen expression, whereas J82COT (grade 4) cells express low levels of Le^x^. These differences in the Le^x^ antigen expression correlate with differences in mRNA expression levels of α1,3/4-fucosyltransferase genes. This suggests that the Le^x^ antigens are promising candidate biomarkers for grading BC. However, the main difference between clinical and in vitro studies should be noted. While in BC patients, the expression of the Le^x^ antigen has been found to increase gradually with cancer progression [167], the cell lines from the most advanced stages have shown a decrease in expression of the Le^x^ antigen in comparison to those cells from earlier stages of the disease [171].

Moreover, Lewis antigen-mediated E-selectin adhesion has been shown to play a role in BC [172]. A titertray assay with immobilized selectins was used to test the adhesion of BC cells isolated from several tumors. The results showed a positive correlation between the number of bound BC cells isolated from several tumors and Le^a^ antigen expression. It suggests that ligands for E-selectin on the surface of BC cells likely bind through α1,4-linked fucose to the penultimate GlcNAc.

Also, increased sialylation of cancer cells often results from the overexpression of the SLe^a^ antigen and the SLe^x^ antigen, which can be detected as terminal epitopes of N-glycans, O-glycans, and glycolipids [173]. Increased serum levels of the SLe^a^ antigen have been associated with higher stage/grade and increased invasiveness of BC. On the other hand, loss or decrease of the SLe^a^ antigen expression in tumor tissues has been associated with a higher atypia grade of the tumor. It has also been shown that expression of the sLex antigen is correlated with shorter 5-year and 7-year survival rates [163].

Finally, BC is a frequent health problem in rural areas of Africa and the Middle East, where *Schistosoma haematobium* is prevalent. It supports a connection between malignant transformation and infection by this parasite, and, importantly, the level of sialylated Lewis antigens is strictly connected to this phenomenon. Infection with *S haematobium* cause an increased expression of SLe^a^ in the majority of benign or pre-malignant BC lesions. It has been shown that *S. haematobium* was responsible for an increased expression of the SLe^a^ antigen in the majority of benign or pre-malignant BC lesions. In addition, *S. Haematobium* eggs show expression of SLe^a^ and SLe^x^ antigens that mimic the glycosylation of human leukocytes. This enables evasion from immune surveillance, easier parasite colonization, and cancer development. This consideration may be of critical value for the early identification of infected patients and for facilitating the development of non-invasive diagnostic tools [139].

## 10. Glycosylation of Extracellular Vesicles in Bladder Cancer

Extracellular vesicles (EVs) are small spherical structures surrounded by a phospholipid bilayer that are released into the intercellular space by almost all cell types [174]. EVs can be found circulating in various body fluids (e.g., blood, cerebrospinal fluid, sperm, saliva, and urine) or in conditioned cell culture media. Based on their size, biogenesis, and molecular composition, EVs are classified into exosomes (30–150 nm in diameter), ectosomes (100–1000 nm), and apoptotic bodies (>1 μm) [175]. The number of released EVs and their molecular composition undergoes constant changes depending on the current state of the parental cell. Bioactive molecules transferred via EVs can then modulate various biological processes in recipient cells, including gene expression, protein biosynthesis, the course of metabolic pathways, and intracellular signaling [176]. Moreover, tumor-derived EVs can participate in angiogenesis, metastasis, multi-drug resistance, apoptosis inhibition, and immunosuppression [177].

The amount of data on BC-derived EV glycosylation is, however, limited. As already mentioned in Section 8, biomarker combinations consisting of ITGA3 and fucose-recognizing UEA lectin using EVs can successfully discriminate BC patients from those with benign lesions and prostate cancer patients [164]. In our recent lectin-based study analyzing the expression of particular glycoepitopes in ectosomes released by normal urothelial HCV-29 and T-24 BC cells, we have observed that the amounts of β1,6-branched tri- and/or tetraantennary complex N-glycans as well α-2,6-linked sialic acids were higher in T-24-derived ectosomes than in HCV-29-derived ectosomes as assessed by immunoblotting [178]. That reflected relationships that were also present in the parental cells. However, flow cytometry analysis has shown that only the levels of ectosomal bisecting GlcNAc and α2,3-linked sialic acids could be considered possible diagnostic BC glycobiomarkers due to their greater amount in T-24-derived ectosomes. Regardless, our study has shown that the glycosylation status of T-24 cells and HCV-24 cells was responsible for the effect that ectosomes released by them had on the viability and motility of the recipient cells. In another study, the antitumor effects of exosomes derived from genetically modified T-24 BC cells expressing glycosyl-phosphatidylinositol-anchored interleukin 2 (GPI-IL-2) have been investigated [179]. Exosomes expressing GPI-IL-2 have been demonstrated to induce the proliferation of T cells and enhance the antigen-specific cytotoxic T-lymphocyte immune response, contrary to exosomes from cells not expressing GPI-IL-2. It suggests that modification of EV cargo with various types of glycans (herein GPI anchor) could be an interesting approach in exosome-based cancer immunotherapy.

## 11. Proteoglycans in Bladder Cancer

**Figure 7:**
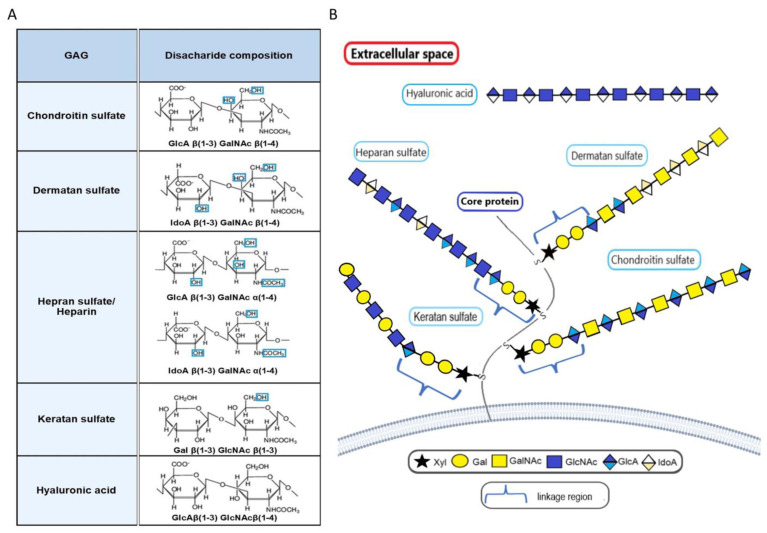
Disacharide composition of GAGs. Heparan sulfate, chondroitin sulfate, keratan sulfate, and dermatan sulfate have a common tetrasaccharide linkage region consisting of xylose (Xyl), two galactose (Gal) residues, and single glucuronic acid (GlcA). Hyaluronic acid consists of alternately appearing N-acetyloglucosamine (GlcNAc) and iduronic acid (IdoA). The figure presents chemical structures of GAGs (**A**) and their schematic visualization (**B**).

The transitional epithelium of the bladder is lined with a layer of glycocalyx composed of glycosaminoglycans (GAGs) and other molecules. GAGs ensure impermeability in the bladder [180], and the damage to the GAG layers may lead to penetration of urine, toxic agents, and bacteria into the deeper layers of the bladder. GAGs represent a type of linear, large heteropolysaccharides. Based on the chemical structure of their repeating disaccharide units that consist of an amino sugar (D-glucosamine that is N-acetylated or N-sulphated, or N-acetyl-d-galactosamine) and either uronic acid (D-glucuronic acid or L-iduronic acid) or galactose, GAGs are classified into different categories: hyaluronic acid (HA), heparin/heparan sulfate, chondroitin sulfate, dermatan sulfate, and keratan sulfate (Figure 7). One or more GAG chains, except for HA, are covalently linked via a tetrasaccharide linkage to the core protein to form highly polyanionic proteoglycans due to the additional sulfate groups present on most of the units.

By acting directly on cellular receptors or through interactions with growth factors, GAGs can regulate multiple cellular processes and are considered essential in cancer biology. Abnormal forms (with altered chain sulphation patterns, molecular size, composition, etc.) and distribution of GAGs have been reported in various cancers. Their influence on the proliferation, apoptosis, adhesion, migration, invasion, and hematogenous metastasis of cancer cells, as well as the induction of angiogenesis and immunosuppression in the tumor microenvironment, have been well documented [181,182,183,184]. Several potential applications of GAGs in cancer treatment have also been reported [185,186].

GAG content is elevated in BC compared to normal urothelium, and its composition is also affected by tumor stage and grade [187,188]. Hyaluronic acid and dermatan sulfate were found to be decreased, while chondroitin sulfate to be increased in invasive cancers. The decrease in the percentage of heparan sulfate was closely correlated with higher-grade tumors. Urinary GAG excretion was also found to increase in parallel with tumor size, stage, and grade [189] and to help follow-up BC patients after transurethral resection [190]. These early observations were confirmed by more recent studies in which tumor grade and stage directly correlated with GAG levels in tissue samples; however, GAG levels did not predict tumor recurrence [191].

In contrast, urinary levels of HA and its degrading enzyme, hyaluronidase (HAase), have been shown to be elevated in BC patients and were indicated as markers for monitoring tumor recurrence and screening (HA-HAase test) [192,193,194]. HA is the major ligand for CD44, which is a multifunctional mediator of cancer progression involved in promoting proliferation, motility, invasiveness, angiogenesis, and chemoresistance [195,196,197]. Interestingly, CD44 expression has been shown to be significantly associated with tumor aggressiveness in BC [117].

HA is synthesized by hyaluronic acid synthase (HAS), which has three isoforms: HAS1, HAS2, and HAS3. In BC cells and tissues, transcript and protein levels of HAS1 were elevated and correlated with bladder tumor recurrence and response to treatment [198,199]. In in vivo and in vitro models, downregulation of HAS1 expression was shown to reduce growth, invasion, and angiogenesis of BC through induction of apoptosis [200].

Similarly, HAS2 and HAS3 have also been shown to promote tumor growth and metastasis [199,201,202]. In addition, HAS2 expression negatively correlated with amylo-α-1,6-glucosidase-4-α-glucanotransferase (AGL), one of the enzymes catalyzing glycogenolysis, which has been described as a tumor growth suppressor and prognostic marker in human BC [203]. It has been shown that patients with high mRNA expression of HAS2 and low AGL following induction of HA synthesis had poor survival rates [204]. Two major HA-binding cell surface proteins, namely CD44 and Hyaluronan Mediated Motility Receptor (RHAMM), are crucial for the rapid growth of BC driven by the loss of AGL [205]. Loss of either CD44 or RHAMM induces apoptosis in BC cell lines with low AGL. In addition, increased expression of HYAL-1 type HAase has been found in BC tissues and has been indicated as an independent predictor of muscle invasion [199,206]. Finally, a thorough meta-analysis has shown that both HA and HAases can be used as biomarkers in the diagnosis of BC [207].

Also, another GAG, chondroitin sulfate, has been found to inhibit the growth of BC cells [208]. One of the carriers of chondroitin sulfate is decorin, a small leucine-rich class 1 proteoglycan consisting of a core glycoprotein with a molecular weight of 40 kDa and a single GAG chain, chondroitin sulfate or dermatan sulfate, which is bound to the N-terminus of the core protein. Decorin functions as a natural inhibitor of pan-tyrosine kinase and negatively regulates tumor growth [209,210]. In BC tissues, the expression of decorin was downregulated compared to that in normal tissue [211,212]. In various cancers, low expression of decorin correlated with poor patient prognosis, lymph node metastasis, and weak response to treatment [210]. The introduction of decorin into BC cells not expressing this protein upregulated TGF-β1 and MMP2 expression via p21 protein, promoted apoptosis and adhesion, and inhibited bladder cancer cell proliferation and metastasis [212,213]. On the other hand, biglycan, which is another small leucine-rich proteoglycan, has been shown to suppress BC cell proliferation and tumor growth [214]. Biglycan levels have been found to increase in advanced stages of cancer in tissue samples and be associated with increased survival of BC patients.

Interestingly, bladder cancer cells have also been shown to express a distinct form of chondroitin sulfate, namely oncofetal chondroitin sulfate (ofCS), which is normally confined to the placenta [215,216]. The carriers of ofCS are syndecan 1 (SDC1) and chondroitin sulfate proteoglycan 4 (CSPG4), proteoglycans that are highly expressed in BC and thus increase the overall amount of ofCS in the tumor [216]. It has been demonstrated that ofCS modulates tumor cell motility by affecting canonical integrin signaling pathways [217]. The presence of urinary ofCS has been shown to correlate with grade and tumor size [218], as well as with resistance to chemotherapy and poorer survival of BC patients [216].

## 12. Manipulation of Glycome as a Therapeutic Tool in Bladder Cancer

As already mentioned in Section 10, genetic engineering gives the possibility to manipulate glycosylation profiles of various cells that can be used in cancer therapy. For instance, multiple carcinomas express extracellular N-glycans, the presence of which negatively correlates with chimeric antigen receptor T (CAR T) cell killing. The knockout GnT-V in pancreatic adenocarcinoma revealed that N-glycans protect tumors from CAR T cell killing by interfering with proper immunological synapse formation and cytokine production leading to decreased cytotoxicity [219]. In the same study, treatment with glucose/mannose analog 2-deoxy-d-glucose (2DG) disrupted the N-glycan cover on tumor cells and enhanced CAR T cell activity. Moreover, 2DG treatment interfered with the PD-1–PD-L1 signaling pathway and increased the survival of tumor-infiltrating CAR T cells in vivo [219]. Therefore, manipulation of the glycosylation profile should be considered in the further development of CAR T therapy, also in the treatment of BC.

Furthermore, core 1 synthase glycoprotein-N-acetylgalactosamine 3-β-galactosyltransferase 1 (C1GALT1) is the key enzyme in the conversion of Tn antigen to T antigen, and its upregulation is observed in BC. Silencing of C1GALT1 suppressed the migratory ability and proliferation of BC YTS-1 cells in vitro and in vivo in YTS-1 inoculated mice [143]. Moreover, miR-1-3p, typically downregulated in urothelial carcinoma, inhibits the transcription of C1GALT1. Artificially induced miR-1-3p overexpression in YTS-1 cells has resulted in their decreased migration and proliferation. It suggests that targeting glycosylation-related enzymes with miRNAs should be considered when designing novel therapies for BC [143].

Another study showed that also manipulation of O-GlcNAc levels might help in disease containment. Melatonin treatment was shown to decrease O-GlcNAc levels which are typically elevated in BC. Melatonin decreased the amount of O-GlcNAc by reducing glucose uptake and availability of GlcNAc donor, i.e., UDP-GlcNAc. This treatment inhibited the proliferation and migration of BC cells through the inhibition of cyclin-dependent-like kinase 5 (CDK5) expression and O-GlcNAcylation of CDK5 [62].

Finally, specific glycan modification may be used to improve the drug delivery process to BC tumors. Intravesical injection of chemotherapeutic drugs such as epirubicin (EPI) is routinely used after transurethral tumor resection. Unfortunately, EPI lacks tumor selectivity and often leads to damage of normal bladder urothelium and other side effects [100]. Because mannose is the most aberrantly expressed glycan on the surface of BC cells, the lectin-drug conjugate has been created by linking concanavalin A (ConA) (mannose-specific lectin) with EPI. This conjugate showed selective cytotoxicity to cancer cells without attacking normal bladder tissues [100].

## 13. Conclusions and Future Perspectives

In this review, we have discussed the role of aberrant glycosylation in BC development and progression. Presented alterations facilitate metastasis formation, immunosuppression, drug and apoptosis resistance, and angiogenesis during tumor progression. A wide range of alterations in glycosylation are observed in the advanced stages of BC. Therefore, it is crucial to compare full glycosylation profiles of early- and advanced-stages of BC to determine how it changes during disease progression.

Nevertheless, the structural complexity of glycans, heterogeneity in glycosylation sites, and the role of epigenetic and environmental factors in glycan structures make a complete characterization of BC glycome a challenge. However, the development of high-performance liquid chromatography (HPLC) and mass spectrometry (MS) enables detailed structural analysis of glycoconjugates. These advanced techniques can be used to analyze not only samples from cell cultures but also tissue and urine samples from BC patients. However, it should be noted that lectins and antibodies are still commonly used to analyze glycosylation, considering the costs of more sophisticated analytical techniques. Additionally, the creation of a BC-related glycosylation database would be useful to analyze the observed alternations of glycan structures, mutations in glycan-related genes, and the role of glycoconjugates in cancer progression.

Recently, several large-scale cancer-related glycoproteomic studies on intact glycopeptides emerged. Analysis of intact glycopeptides instead of isolated glycans allows the identification of glycosylated proteins, including those expressing TACAs. Moreover, such studies can precisely determine glycosylation sites within proteins/peptides. It also creates diagnostic and/or therapeutic opportunities, for instance, involving genetic engineering. Elimination or introduction of particular glycosylation sites could make it possible to regulate the function of chosen carcinogenesis-related proteins by altering their glycosylation status. It has already been shown for ovarian cancer that integrated glycoproteomic analysis of tumor tissues enables subtyping (clustering) and outcome prediction for high-grade tumors [220,221]. Nevertheless, glycoproteomic strategies still battle some technical limitations. Glycopeptide signals are more difficult to recognize than signals from naked peptides due to their microheterogeneity. Moreover, working in the data-dependent acquisition (DDA) is limited to the MS2 level of tandem MS1/MS2 analysis. It is impossible to identify all glycoforms present in the sample using only MS1 scans due to the high peak intensities. However, recently, efficient software tools have been developed. One of them is pGlycoQuant, which applies a deep learning model that reduces missing values by 19–89% [222].

Targeting aberrant glycosylation and using it as a diagnostic and therapeutic tool in BC is strongly supported by studies presented in this review. For instance, the use of glycosyltransferase inhibitors or glycosidases in BC treatment could limit the side effects of classical chemotherapy, especially affecting the kidneys and liver. Moreover, BC cell lines derived from invasive tumors displayed increased expression of FUT6 and FUT7. It gives an opportunity to target not only glycosylation-related enzymes in the therapy of BC but also in diagnosis. Diagnosis of BC still relies on invasive methods such as cystoscopy and TURBT. Therefore, focusing on TACAs differential expression may lead to the establishment of new diagnostic tools with higher specificity for early detection, prognosis, and grading BC.

**Figure 8:**
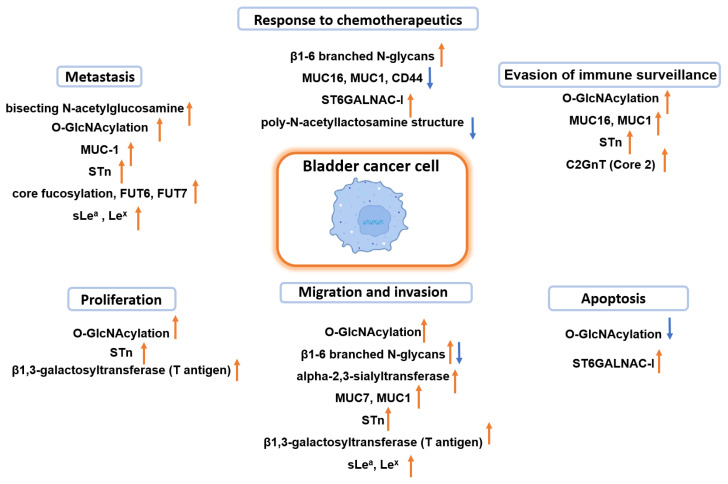
Summarized role of altered glycosylation in bladder cancer progression.

In summary, glycosylation status plays a vital role in BC progression (Figure 8). In recent years we observed incredible progress in understanding the effects caused by altered glycosylation. Numerous studies have focused on glycans as promising biomarkers and therapeutic tools in BC, highlighting the importance of glycobiology in cancer-related studies.

## Data Availability

Not applicable.

## Abbreviations

β4GalT1	β1:4-galactosyltransferase-1
2DG	2-deoxy-d-glucose
ADCC	antibody-dependent cellular cytotoxicity
AGP	α-1-Acid glycoprotein
BC	Bladder cancer
BCG	Bacillus Calmette-Guérin
C1GnT	Core 1 β1,3-galactosyltransferase
CAR T	Chimeric antigen receptor T
CD44	Cluster of Differentiation 44
CDK5	Cyclin-dependent-like kinase 5
CRT	Calreticulin
CSCs	Cancer stem cells
CTCs	Circulating tumor cells
CTLs	Cytotoxic T-lymphocytes
DCs	Dendritic cells
ECM	Extracellular matrix
EGF	Epidermal growth factor
EMT	Epithelial-mesenchymal transition
EPI	Epirubicin
EVs	Extracellular vesicles
FUTs	Fucosyltransferases
GlcNAc	N-acetylglucosamine
GnT III	N-acetylglucosaminyltransferase III
GnT IV	N-acetylglucosaminyltransferase IV
GnT V	N-acetylglucosaminyltransferase V
GPI	Glycosylphosphatidylinositol
GPI-IL-2	Glycosyl-phosphatidylinositol-anchored interleukin 2
HLA	Human leukocyte antigen
Igs	Immunoglobulins
ITGA3	α3 integrin subunit
Le^a^	Lewis^a^
Le^b^	Lewis^b^
Le^x^	Lewis^x^
Le^y^	Lewis^y^
MIBC	Muscle-infiltrating bladder cancer
Neu5Ac	N-acetyl-neuraminic acid
Neu5Gc	N-glycolyl-neuraminic acid
NK	Natural killer
NMIBC	Non-muscle-infiltrating bladder cancer
OGT	O-GlcNAc transferase
PHAL	Phaseolus vulgaris leucoagglutinin
Sia	Sialic acids
SLex	sialyl-Lewis X
ST antigen	Sialilated T antigen
ST6GALNAC-I	ST6 N-acetylgalactosaminide α-2,6-sialyltransferase 1
TACA	Tumor-associated carbohydrate antigens
TGFβ	Transforming growth factor β
TURBT	Transurethral resection of bladder tumor
UC	Urothelial carcinomas
UEA	*Ulex europaeus* agglutinin
